# Editorial: Ureteropelvic Junction Obstruction (UPJO) in Infants

**DOI:** 10.3389/fped.2017.00148

**Published:** 2017-06-26

**Authors:** Ricardo González

**Affiliations:** ^1^Pediatric Surgery and Urology, Kinder- und Jugendkrankenhaus AUF DER BULT, Hanover, Niedersachsen, Germany

**Keywords:** hydronephrosis, ureteropelvic junction obstruction, surgical management, laparoscopic pyeloplasty, infants

**Editorial on the Research Topic**

**Ureteropelvic Junction Obstruction (UPJO) in Infants**

The intention of this Research Topic was to attract articles expressing different points of view on the current approach to evaluate and manage ureteropelvic junction (UPJ) obstruction in infants. Six groups have contributed papers that were accepted for publication. One article addresses the problem of grading hydronephrosis in infants, and another article addresses the controversial topic of the need and efficacy for prophylactic antibiotics when hydronephrosis is detected prenatally. The remaining three papers address the surgical management of the obstructed infant UPJ.

Between the first description of an operation to correct an ureteropelvic junction obstruction (UPJO) by Trendelenburg in 1886 (1) and the 1970s, little changed in the diagnosis and management of this condition. Patients usually presented with abdominal or flank pain, the diagnosis was established by excretory or retrograde pyelography and treatment was surgical by means of an open pyeloplasty.

With the advent of fetal ultrasonography (US), pediatricians and urologists were confronted by an increasing number of babies and children with asymptomatic dilation of the upper urinary tract. The initial over aggressive approach of early intervention was tempered by the realization that the upper tract dilatation in many cases improved or resolved spontaneously with time (2).

The work of many investigators, notably the group in London, helped define the prognosis of untreated patients based on the findings on US. They emphasized the prognostic value (based on preservation of individual renal function an eventual need for surgery) of the anteroposterior diameter of the renal pelvis (AP diameter) (2). Nuclear renography, done principally to determine the function of each kidney was an indispensable additional study. Although this work was reported repeatedly in oral presentations and congresses, published data validating the prognostic value of the AP diameter in peer reviewed journals are lacking. Nevertheless in the last 15 years or so, in most centers, the AP diameter as well as the presence of decreased function of the affected kidney below 40% have been considered the main parameters to indicate surgical correction.

A shortcoming of emphasizing the AP diameter is that the degree of calyceal dilatation and the thinning of the parenchyma may pose a greater risk of function loss than the simple measurement of the AP diameter. Other grading systems such as the one proposed by the Society for Fetal Urology (SFU) addressed this issue but also lack validation and may be more subjective (3). Thus, a grading hydronephrosis that can help define the indications for surgery and that can be validated would be extremely valuable for clinicians. An article in this collection proposes a refinement of the SFU grading system that includes important parenchymal parameters. It is hoped that this proposed system will be used by other groups to test its validity and applicability in clinical practice (Kelley et al.).

A widespread clinical practice has been the administration of antibacterial prophylaxis in all newborns with antenatally detected urinary tract dilatation. Also in this collection, an article addresses this topic (Braga et al.). The authors point out the increase likelihood of urinary tract infection in infants with high grade hydronephrosis and present a proposal to study this topic.

The remaining three articles in this collection address the surgical repair of UPJO in children. Piaggio et al. conducted a randomized prospective study comparing open (OP) versus laparoscopic pyeloplasty (LP) in children in two institutions. They concluded that surgical time was significantly longer in LP than in OP group but length of hospitalization and postoperative analgesia requirement were less in the LP group. At a mean follow-up of 58 months, there were no failures in either group.

Ludwikowski et al. reported their highly successful method to perform laparoscopic pyeloplasty (83% complete resolution of hydronephrosis) in a group of 24 infants with a mean age of 7.9 months, a mean weight of 7.4 kg, and a mean follow-up of 18 months undergoing LP.

A group from Spain reported high success with retrograde dilatation of the UPJO in 16 infants using an angioplasty cutting balloon (Parente et al.). The results of this innovative and minimally invasive method await replication and validation from other centers before it can be recommended for widespread use.

The final paper by Bansal et al. addresses the minimal follow-up needed after pediatric pyeloplasty. Based on their experience the authors recommend a minimum of 3 years or whenever symptoms of possible obstruction are present (Bansal et al.).

In all this collection updates, our knowledge of pediatric UPJO and presents new ideas for future research.

Although the results of OP and laparoscopic repair are similar, the transperitoneal approach done using an umbilical port and two 3-mm working ports has unquestionable cosmetic advantage over OP. I wish to thank all contributors to this Research Topic for their interest and excellent contributions.

## Author Contributions

The author confirms being the sole contributor of this work and approved it for publication.

## Conflict of Interest Statement

The author declares that the research was conducted in the absence of any commercial or financial relationships that could be construed as a potential conflict of interest.
